# Bovine coronavirus in naturally and experimentally exposed calves; viral shedding and the potential for transmission

**DOI:** 10.1186/s12985-016-0555-x

**Published:** 2016-06-13

**Authors:** Veslemøy Sunniva Oma, Madeleine Tråvén, Stefan Alenius, Mette Myrmel, Maria Stokstad

**Affiliations:** Department of Production Animal Clinical Sciences, Norwegian University of Life Sciences, Ullevålsvegen 72, 0454 Oslo, Norway; Department of Clinical Sciences, Swedish University of Agricultural Sciences, 75007 Uppsala, Sweden; Department of Food Safety and Infection Biology, Norwegian University of Life Sciences, Ullevålsvegen 72, 0454 Oslo, Norway

**Keywords:** BCoV, BCV, Experimental infection, Clinical signs, Virus shedding, Transmission potential, Viremia, Biosecurity, Virus quantification, RT-qPCR, Virus isolation

## Abstract

**Background:**

Bovine coronavirus (BCoV) is a widely distributed pathogen, causing disease and economic losses in the cattle industry worldwide. Prevention of virus spread is impeded by a lack of basic knowledge concerning viral shedding and transmission potential in individual animals. The aims of the study were to investigate the duration and quantity of BCoV shedding in feces and nasal secretions related to clinical signs, the presence of virus in blood and tissues and to test the hypothesis that seropositive calves are not infectious to naïve in-contact calves three weeks after BCoV infection.

**Methods:**

A live animal experiment was conducted, with direct contact between animal groups for 24 h as challenge procedure. Four naïve calves were commingled with a group of six naturally infected calves and sequentially euthanized. Two naïve sentinel calves were commingled with the experimentally exposed group three weeks after exposure. Nasal swabs, feces, blood and tissue samples were analyzed for viral RNA by RT-qPCR, and virus isolation was performed on nasal swabs. Serum was analyzed for BCoV antibodies.

**Results:**

The calves showed mild general signs, and the most prominent signs were from the respiratory system. The overall clinical score corresponded well with the shedding of viral RNA the first three weeks after challenge. General depression and cough were the signs that correlated best with shedding of BCoV RNA, while peak respiratory rate and peak rectal temperature appeared more than a week later than the peak shedding. Nasal shedding preceded fecal shedding, and the calves had detectable amounts of viral RNA intermittently in feces through day 35 and in nasal secretions through day 28, however virus isolation was unsuccessful from day six and day 18 from the two calves investigated. Viral RNA was not detected in blood, but was found in lymphatic tissue through day 42 after challenge. Although the calves were shedding BCoV RNA 21 days after infection the sentinel animals were not infected.

**Conclusions:**

Prolonged shedding of BCoV RNA can occur, but detection of viral RNA does not necessarily indicate a transmission potential. The study provides valuable information with regard to producing scientifically based biosecurity advices.

**Electronic supplementary material:**

The online version of this article (doi:10.1186/s12985-016-0555-x) contains supplementary material, which is available to authorized users.

## Background

Bovine coronavirus (BCoV) is an important livestock pathogen with a high prevalence worldwide. The virus causes respiratory disease and diarrhea in calves and winter dysentery in adult cattle. These diseases result in substantial economic losses and reduced animal welfare [1]. One way of reducing the negative consequences of this virus is to prevent virus transmission between herds. Inter-herd transmission is possible either directly via transfer of live animals [2, 3], or indirectly via contaminated personnel or equipment [4]. Measures to prevent virus spread between herds must be based upon knowledge of viral shedding, the potential for transmission to susceptible animals and the role of protective immunity. Several observational studies have been published on BCoV shedding in feces of diarrheic calves and after transportation to feedlots [3, 5–10]. However, relatively few studies on BCoV pathogenesis with emphasis on transmission potential under controlled conditions have been published.

BCoV belongs to the genus *Betacoronavirus* within the family *Coronaviridae,* also including the closely related HCoV-OC43, which causes respiratory infections in humans, and the human pathogens SARS-CoV and MERS-CoV [11–13].

BCoV consists of one serotype with some antigenic variation between different strains [14, 15]. Acutely infected animals develop antibodies that persist for a long period, possibly for several years [16–18]. However, the protective immunity is shorter and incomplete. In two experimental studies, infected calves were not protected against reinfection with a different BCoV strain three weeks after the first challenge, but did not develop clinical signs [19, 20].

BCoV is transmitted via the fecal-oral or respiratory route [15]. It infects epithelial cells in the respiratory tract and the intestines; the nasal turbinates, trachea and lungs and the villi and crypts of the small and large intestine, respectively [21, 22]. Replication leads to shedding of virus in nasal secretions and in feces. Important factors for the pathogenesis are still not fully explored, such as how the virus infects enterocytes shortly after introduction to an animal. Viremia has been detected in one study by Park et al. [21]. Clinical signs range from none to severe, and include fever, respiratory signs and diarrhea with or without blood [1, 15]. As the time of infection is usually unknown and laboratory diagnostics are usually not performed, occurrence of clinical signs is the most relevant parameter to relate to viral shedding. The majority of experimental studies have used BCoV inoculation as challenge procedure, which may influence clinical signs and viral shedding, and thereby the transmission potential compared to natural infection. It has been hypothesized that BCoV can cause chronic subclinical infections which could be an important virus source [15]. Kapil et al. documented viral antigen in the small and large intestines of infected calves three weeks post inoculation [23]. Crouch et al. found that ten cows were shedding BCoV-immune complexes in the feces for 12 weeks [24]. It is, however, difficult to establish whether there is true persistence of virus, or reinfection of partially immune animals and whether these animals represent a risk to other animals. There is a lack of experimental studies investigating viral shedding pattern for longer periods than two weeks, with sensitive detection methods. Viral load and infectivity also needs to be determined. This is of high practical relevance, since the farmers need guidance on biosecurity in trade and transport of live animals.

The current study was conducted to fill prevailing gaps in the knowledge on fundamental aspects of BCoV infection. The specific aims were to:

1. study the duration and quantity of BCoV shedding in feces and nasal secretions, related to clinical signs in calves.
2. study the presence of viremia and persistence of virus in lymphatic, intestinal and lung tissue.
3. test the hypothesis that seropositive calves are not infectious to naïve in-contact calves three weeks after BCoV infection.

## Methods

### Study design

**Fig. 1:**
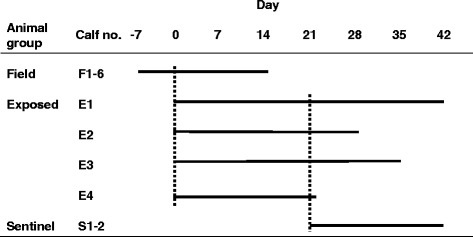
Timeline of the experiment. The solid lines symbolize the timespan when the calves participated in the experiment. The dashed lines symbolize commingling of the indicated animals for 24 h; e.g. the field group arrived at the research facility on day −7, commingled with the exposed group day 0 and left the research facility day 14. The calves in the exposed group were sequentially euthanized from day 22 to day 42. The Sentinel group arrived at the research facility day 21 and commingled with the Exposed group the following 24 hours

A live animal experiment with the natural host was conducted. The experimental units were groups of calves and the intervention consisted of direct contact with BCoV-infected animals. The primary outcome was clinical signs, and the secondary outcome was presence of BCoV RNA and BCoV antibodies. Three experimental groups were included; the Field group (FG, *n* = 6) that was naturally infected with BCoV, the naïve Exposed group (EG, *n* = 4) and the naïve Sentinel group (SG, *n* = 2). An overview of the study design is shown in Fig. 1.

### Animals, housing and husbandry

#### Animals

Twelve BCoV seronegative weaned bull calves between six and twelve weeks of age were included, seven were Swedish red and white, four were Swedish Holstein and one Swedish mountain breed. They originated from two dairy herds, initially negative for antibodies to BCoV in milk from primiparous cows. The calves were allocated to groups according to herd of origin and day of arrival. The sequence of euthanasia of the EG and SG calves was random, determined by drawing of lots.

#### Natural outbreak of winter dysentery

FG originated from a herd that was in an early phase of a winter dysentery outbreak. When FG was transported to the research facility, the calves showed mild signs of respiratory disease. Two days later, a severe outbreak confirmed by RT-PCR and serology to be caused by BCoV with bloody diarrhea and reduction in milk production, took place in the herd.

#### Research facility

The experiment was conducted at the stationary clinic at the Department of Clinical Sciences at the Swedish University of Agricultural Sciences. The facility was closed for other animals during the experiment, and had restricted admission for people. Personnel used designated clothing, and had no contact with other cattle the same day. Each group was housed in separate pens within the same room. Due to the type of facility and design of the study, acclimatization period was not possible for any of the groups. Clinical examinations and sampling were consistently done in the order SG, EG and FG.

#### Challenge procedure

To mimic standard managerial conditions, direct contact was chosen as challenge procedure for both EG and SG. The commingling was done by moving EG into the other two groups’ pens for 24 h.

#### Refinement and treatment procedures

Efforts were made to minimize the stress and discomfort for the animals involved. The calves were kept group-wise in pens with straw bedding, were fed a commercial calf concentrate twice daily and had access to haylage ad libitum. The animals were monitored by a trained animal technician and a veterinarian at least three times a day. Indications for antibiotic treatment (30 000 IU procaine benzyl penicillin/kg bodyweight/day i.m. for five consecutive days) were abnormal sounds on lung auscultation or prolonged high temperature. Indication for treatment with a non-steroidal anti-inflammatory drug (Metacam vet, Boehringer Ingelheim Vetmedica, Germany) was severe depression, and oral fluid with electrolytes was to be given to moderately dehydrated animals. Euthanasia was achieved by i.v. injection of pentobarbital (Euthasol vet., Le Vet, Netherlands).

#### Clinical score

Daily clinical examinations were performed by a veterinarian and clinical signs were scored as presented in Table 1 (modified after Hägglund et al. [25, 26] and Silverlås et al. [27]). A score above two on three consecutive days was categorized as mild clinical disease; a score above six on three consecutive days as moderate disease and a score above eleven was categorized as severe clinical disease.

**Table 1:** Clinical scoring system

Score	Respiratory rate (breaths/min)	Fever	Cough	Nasal discharge	Demeanor	Fecal consistency
0	≤49	≤39,5	No cough observed	Normal	Bright, alert	Normal
1	50–54	39,6–39,9	Sporadic cough	Serous or mucous	Mildly depressed	Pasty
2	55–64	40–40,4	More than one sporadic cough every 10 min of observation	Mucopurulent or purulent	Moderately depressed	Runny
3	65–74	>40,5	–	–	Severely depressed	Watery
4	75–85	–	–	–	–	Runny or watery with blood

The score from each category was added to give a daily clinical score for each of the calves in the experiment

### Collection of material

Nasal swab specimens and fecal samples from FG were collected approximately every third day from day −4 (D-4) to D14. From EG, nasal swabs and fecal samples were collected every day from D0 to D25 and then every third day until D35. Nasal swabs from SG were collected D24, D27 and D29. The nasal specimens were collected by rotating a flocked ESwab™ (Copan, Brescia, Italy) approximately five cm inside one of the calf’s nostrils. The specimens were frozen and stored at −70 °C before further processing. Blood was drawn from the jugular vein upon arrival and D1, D2, D3, D5, D7, D9, D11, D14, D21, D35 and D41 using sterile evacuated tubes with and without EDTA-anticoagulant. The EDTA-blood was centrifuged and the cell fractions were stored separately at −80 °C before further processing. Sera were stored at −20 °C until analyzed. Tissue samples from lung, medial retropharyngeal and mesenteric lymph nodes, ileum, and colon were stored in RNA-later at −20 °C.

### Antibody ELISA

Serum samples were analyzed for anti BCoV IgG by Svanovir BCV-Ab (Boehringer Ingelheim Svanova, Uppsala, Sweden) according to the manufacturer’s instructions. Samples from SG were also tested for antibodies to bovine respiratory syncytial virus (BRSV) by Svanovir BRSV-Ab (Boehringer Ingelheim). The optical density (OD) at 450 nm was measured and corrected by subtracting the OD for the negative control. Percent positivity (PP) was calculated as (sample OD/positive control OD) × 100, and a PP-value of <10 was regarded as negative.

### Extraction of RNA and RT-qPCR

Fecal samples (diluted 1:10 in PBS) and nasal swab specimens were centrifuged at 9700 x *g* for 10 min. RNA was extracted from 140 μl supernatant and 140 μl plasma by QIAamp Viral RNA Mini QIAcube kit (Qiagen, Hilden, Germany), eluted in 50 μl and frozen at −80 °C. RNA from blood cell fractions from calf E4 on D5 and calf E3 on D7 was extracted with Qiazol (Qiagen) and chloroform phase separation mixed with 70 % ethanol (1:1) and purified using RNeasy Mini Kit column (Qiagen), while RNA was extracted from 30–50 mg tissue samples, using RNeasy Plus Universal Mini Kit (Qiagen). RT-qPCR was performed using RNA UltraSense™ One-Step Quantitative RT-PCR System (Invitrogen, MA, USA). Two microliters of RNA was added to a 18 μl reaction volume containing 200 nM each of forward and reverse primers and 250 nM TaqMan probe [28]. The thermal profile included an RT step with 30 min at 55 °C followed by 95 °C for 2 min. Thereafter, 40 cycles with 15 s at 95 °C and 60 s at 60 °C were conducted. The RT-qPCR was performed on a Stratagene Mx3005p™ (Agilent Technologies, CA, USA) and a positive and a negative control were included in each run. In order to evaluate inhibition of the RT-qPCR, RNA extract from some fecal samples were diluted 1:10 and compared to undiluted RNA. The Ct-values in these samples suggested negligible levels of inhibitors. Inhibitors in plasma and cell extracts were evaluated by spiking with mengovirus RNA. Comparison of Ct-values showed that plasma had no negative effect, while the cell fractions had an inhibitory effect, giving an increase of one Ct-value.

### Virus quantitation

In order to estimate the number of BCoV viral RNA copies (VRC) in the clinical samples, a standard curve was prepared using tenfold dilutions of a plasmid containing the BCoV target sequence. Aliquoted BCoV RNA was used as a calibrator and included in every RT-qPCR plate to adjust for inter plate variation. The number of VRC in the clinical samples was calculated using the formula:

$$ {Q}_S={Q}_C\ast {10}^{\frac{C{t}_S-C{t}_C}{m}} $$

Where *Q*_*s*_ *= viral RNA copies in sample, Q*_*c*_ *= viral RNA copies of calibrator, Ct*_*s*_ *= Ct value of sample, Ct*_*c*_ *= Ct value of calibrator* and *m = slope of the standard curve.*

The standard curve covered the range from 10.8 to 1.08 × 10^10^ plasmid copies, and showed a strong linear relationship with a high coefficient of determination (R^2^ = 0.996) and a high amplification efficiency (96.5 %). The limit of quantification (LOQ) for the plasmid was 10.8 copies which represented 3.6 log_10_ BCoV VRC per nasal swab and ml plasma, 4.6 log_10_ VRC/g feces and 4.2 log_10_ VRC/g tissue.

### Virus isolation

Virus infectivity was tested by virus isolation from nasal swabs from E1 and E3 between D3 and D28 (D3, D6, D7, D8, D10, D13, D18, D23 and D28). The swab supernatants were diluted 1:25 in Dulbecco’s Modified Eagle Medium (DMEM, Thermo Fisher Scientific, Paisley, Scotland), filtered through a 0.8 μm filter (Sartorius Stedim Biotech, Goettingen, Germany) and added to a monolayer of 4-days-old human rectal tumor cells (HRT-18G, ATTCC CRL-11663) in a 24-well plate. In addition, infective virus was titrated from one nasal swab supernatant using two-fold endpoint dilutions in a 96-well plate. After 1 h incubation at 37 °C, the inoculum was replaced with DMEM with 1 % fetal calf serum and antibiotics (5000 IU penicillin and 5 mg streptocillin/ml). After two days at 37 °C and 5 % CO_2_, the cells were fixed with Intracellular Fixation buffer (eBiosience, CA, USA) and stained with 1:80 dilution of monoclonal mouse anti-coronavirus antibody labelled with fluorescein isothiocynate (BioX Diagnostics, Rochefort, Belgium) and DAPI nuclear counterstain (Thermo Fischer Scientific). The wells were observed under a fluorescent microscope for antigen positive cells.

## Results

### Clinical outcome

**Fig. 2:**
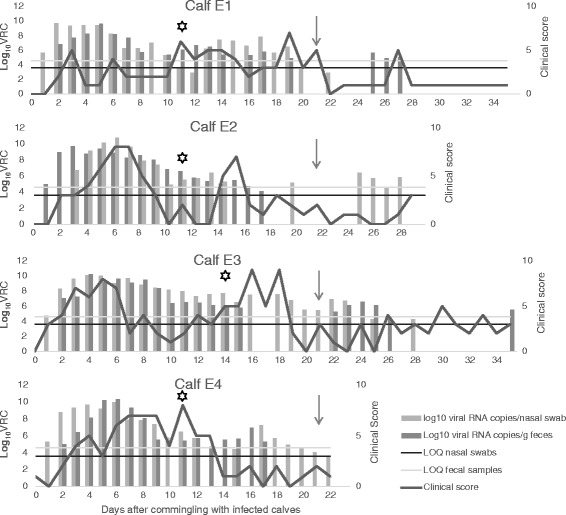
Clinical score and viral shedding in BCoV infected calves. Number of BCoV viral RNA copies (VRC) per nasal swab and per gram of feces collected from calves in the Exposed group (EG) from day 0 (the day of commingling with the Field group) through day 35. Limit of quantification (LOQ) in nasal swab specimens and fecal samples is indicated with horizontal lines. The values under LOQ are extrapolated and less accurate. A star indicates the day of seroconversion (percent positivity > 10) to BCoV and the arrow indicates the day of commingling with the Sentinel group (day 21). The clinical scores are calculated based on daily registrations of clinical signs from the calves in EG

An overview of clinical signs in all groups is presented in Table 2. Five out of six FG calves showed mild clinical disease. EG’s daily clinical scores are shown in Fig. 2. Three out of four EG calves showed mild disease, and one calf moderate clinical disease. SG did not develop clinical signs that were categorized as disease in the clinical scoring system. However, both calves had some days with intermittent nasal discharge and sporadic cough and S1 had a few days with intermittently runny feces. Blood-tinged diarrhea or nasal discharge was not observed in any of the groups.

**Table 2:** Key clinical signs and treatment during an experiment with BCoV infected calves

Animal group	Calf no.	Peak rt^a^ (°C)	Number of days with				Peak clinical score	Days with clinical score >6	Day of treatment initiation^d^
			depression	diarrhea^b^	nasal discharge^c^	respiratory rate ≥65			
Field	F1	40,3	0	3	1	0	5	0	−1
	F2	39,8	3	2	1	0	8	2	3
	F3	40,1	2	0	7	0	7	1	−5^e^
	F4	40,6	1	0	4	1	7	2	2
	F5	39,7	2	13	1	2	6	0	-
	F6	39,2	1	0	4	0	5	0	-
Exposed	E1	39,5	7	0	2	3	7	1	-
	E2	39,8	4	1	7	1	8	3	7
	E3	40,2	8	1	19	2	9	5	5 and 18
	E4	39,9	6	1	8	0	8	4	-
Sentinel	S1	39,2	1	4	3	0	5	0	-
	S2	39,4	2	0	3	0	4	0	-

The calves were exposed to BCoV in the field (F1-6), were exposed to F-animals (E1-4) or exposed to E-animals (S1-2). ^a^Peak rectal temperature (rt) ^b^Runny to watery stools were considered diarrheic. ^c^ Mucopurulent or purulent nasal discharge (nasal discharge score =2). ^d^ Five days of i.m. treatment with 30 000 IU procaine benzylpenicillin was initiated on indicated day. ^e^ Calf F3 was treated for six days

### Serology

All calves tested negative for antibodies to BCoV at the beginning of the trial. At D14 all calves in FG and EG had seroconverted (Additional file 1: Table S1). The SG was still seronegative to BCoV D42 and did not show an increase in titer for antibodies to BRSV.

### Viral RNA in blood

BCoV RNA was not detected in any of the blood samples analyzed.

### Nasal shedding of viral RNA

**Fig. 3:**
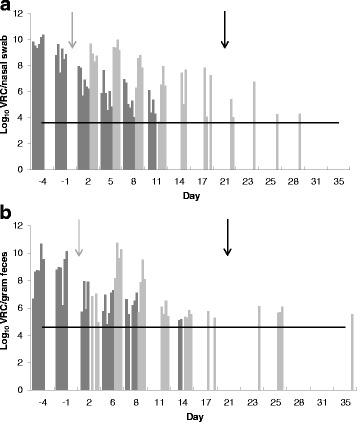
Log_10_ viral RNA copies (VRC) of BCoV per nasal swab (**a**) and gram feces (**b**). Shedding of BCoV from calves in the Field group (FG) (dark grey) and in the Exposed group (EG) (light grey). Grey arrow; day of EG and FG commingling. Black arrow; day of Sentinel group and EG commingling. The horizontal lines show the limit of quantification of VRC

The nasal shedding of BCoV RNA from FG and EG is presented in Fig. 3a, and Fig. 2 shows EG calves’ individual shedding. Briefly, FG was shedding BCoV RNA D-4 through D11, and in EG all swabs were positive from D1 through D12, and at least one out of four calves was positive through D28 (Fig. 3a). Two calves were positive in nasal swabs with a concentration of 5.4 log_10_ and 4.0 log_10_ VRC/swab the day of commingling with SG. None of the nasal swabs from SG were positive.

### Fecal shedding of viral RNA

Fecal shedding of BCoV RNA in FG and EG is shown in Fig. 3b, and the individual shedding from EG in Fig. 2. Viral RNA was detected in fecal samples from FG between D-4 and D14. Fecal samples from EG were negative D0 and D1. At least two out of four calves were positive every day from D2 through D17 and BCoV RNA was intermittently detected through D35. After D14, three calves had a period of four to six days with negative results, before they again started shedding BCoV RNA for three to five days (Fig. 2).

### Association between PCR positivity and clinical signs

**Fig. 4:**
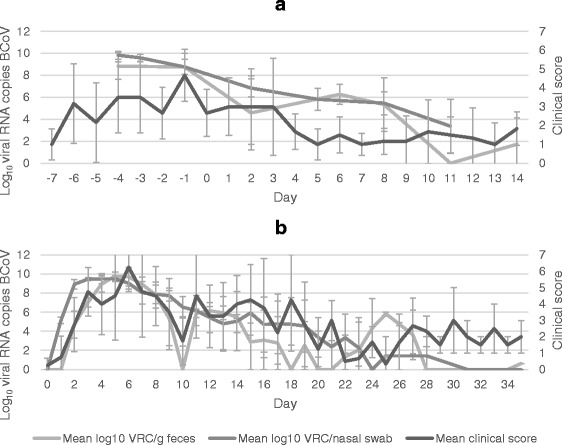
Clinical score and viral shedding in calves exposed to BCoV. Mean clinical score and mean log_10_ viral RNA copies (VRC) of BCoV per nasal swab and gram feces from calves in the Field group (FG) (**a**) and Exposed group (**b**)

**Fig. 5:**
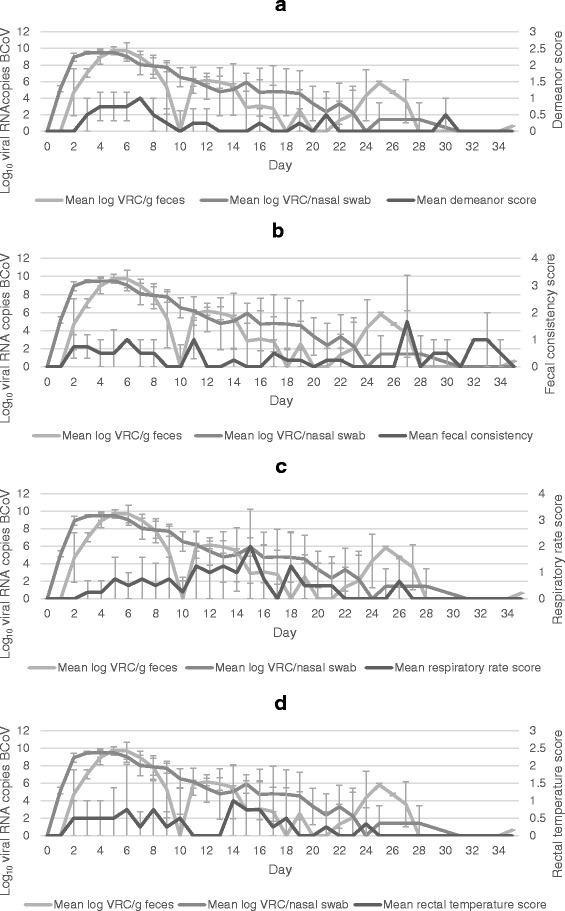
Association between viral shedding and scoring of clinical signs. Mean daily shedding of BCoV and scoring of demeanor (**a**), fecal consistency (**b**), respiratory rate (**c**) and rectal temperature (**d**) of calves in the Exposed group after exposure to BCoV infected calves. The shedding is shown as mean log_10_ viral RNA copies (VRC) of BCoV per nasal swab and gram feces

The association between BCoV PCR results and selected clinical signs is shown in Figs. 4 and 5. The overall clinical score showed good correlation with detection of BCoV RNA. General depression and cough were the individual scores that showed the best association with BCoV RNA shedding. The highest mean respiratory rate and rectal temperature appeared more than a week later than the peak shedding.

**Fig. 6:**
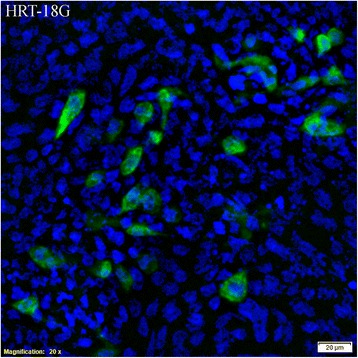
HRT-18G cells infected with BCoV from a nasal swab. The cells were infected with supernatant from a nasal swab taken from calf E3 six days after exposure to BCoV. The cells are stained with anti-coronavirus antibodies labelled with fluorescein isothiocynate and DAPI nuclear counterstain

### Viral RNA in tissues

Viral RNA was detected in lymph nodes from the EG calves euthanized three, four, five and six weeks after infection (Table 3). Viral RNA was also detected in ileum and colon from the animals euthanized five and six weeks after infection, but not in lung tissue.

**Table 3:** Log_10_ viral RNA copies of BCoV per gram tissue

Days post exposure	Calf	Medial retropharyngeal lymph node	Mesenteric lymph node	Lung	Ileum	Colon
22	E4	6.9	6.3	Not done	Not done	Not done
28	E2	6.7	Negative	Not done	Not done	Not done
35	E3	Negative	5.0	Negative	6.0	5.2
42	E1	6.2	7.4	Negative	7.0	6.0

Tissue samples from lymph nodes, lung, Ileum and Colon were harvested from exposed group calves euthanized at the indicated number of days after exposure to field group calves. The number of viral RNA copies (VRC) of BCoV was quantified with RT-qPCR and the limit of quantification was 4.2 log_10_ VRC/g tissue

### Virus isolation

Virus was isolated from nasal swabs from calf E1 on D3 and from E3 in the period D3 to D13. A photograph of infected cells is shown in Fig. 6. The titer of infective BCoV in the nasal swab was 2560 per 50 μl swab medium (1 ml in total) corresponding to 4.7 log_10_ infective particles in a swab containing 9.8 log_10_ VRC, giving a total to infective particles ratio (T/I) of 5 log_10_.

## Discussion

The present study showed that calves infected with BCoV shed viral RNA for five weeks, and harbored viral RNA in intestinal tissues and lymph nodes even longer. Interestingly, contact with these calves three weeks after challenge, when the clinical condition had improved and the calves had seroconverted, did not lead to infection in sentinel calves and virus isolation was not possible from calves shedding viral RNA at this time point.

In concordance with other studies [18, 29], all EG calves became BCoV positive shortly after contact with infected calves and shed viral RNA continuously for two weeks. This supports that introduction of BCoV into a naïve population leads to a high basic reproduction number (R_0_). R_0_ depends on the duration of the infectious period, the number of exposed susceptible individuals and the probability of a susceptible individual to be infected. In herds and transportation systems where cattle from different herds are commingled, the risk of virus transmission is high.

The detection of BCoV RNA in nasal swabs from naïve calves in EG shortly after exposure might be due to passive inhalation of virus excreted by the FG, or to virus replication in the respiratory tract. Since the viral load in the nasal swabs from EG exceeded that of FG at D2, the study confirms that BCoV replicated massively in the airways of EG calves already at D2. Fecal shedding started later than nasal shedding which is in concurrence with other studies [30]. Saif and colleagues found that when inoculating calves intranasally, BCoV was first detected in nasal epithelial cells and secondly in feces. In contrast, in calves inoculated orally, fecal detection of BCoV preceded detection in nasal swab specimens. They concluded that the infection route could determine the sequence of infection of the respiratory and intestinal tract [22]. The present study supports that the respiratory route is the most common infection route when calves are naturally infected by direct contact. With indirect virus spread, the fecal-oral route could be more common.

Nasal swabs were more often positive for BCoV than fecal samples in this trial, most likely due to a higher limit of detection for BCoV in feces than in nasal swabs. For diagnostic purposes, nasal swab specimens therefore seem advantageous to fecal samples for virus detection in calves with suspected BCoV related disease.

Moving and commingling are associated with stress, which has been found to affect the intestinal immune system [31]. It is possible that stress increased the BCoV RNA shedding observed in the EG calves after introduction of the sentinel calves. Buying and selling of calves often involve extended transportation and commingling with susceptible cattle. The stress response, and a possible increased fecal shedding of virus, would probably be higher under field conditions.

In the acute stage of the infection, the agreement between positive PCR results and clinical score was relatively high. Three weeks after exposure to BCoV, the clinical signs and detection of viral RNA varied more independently. In an experiment with porcine deltacoronavirus, the severity of the clinical signs did not correlate with the shedding of virus in conventionally reared piglets, only in gnotobiotic piglets [32]. This indicates that secondary pathogens and changes in microbiota are important for disease development and clinical signs. The present study supports that after the acute stage of disease other factors than virus replication are important for clinical signs; for instance secondary bacterial infections.

Although the sentinel calves did not get infected with BCoV, they showed sporadic unspecific signs during the trial, but below the mildest category “mild disease” in the clinical scoring system. Since acclimatization was not possible, the calves changed environment including feeding routines when enrolled in the experiment, which could cause the signs observed. Other infectious agents could also have been present, and if so, most likely less virulent pathogens. Bovine virus diarrhea virus and bovine herpesvirus 1 are not present in Sweden [33], and the sentinel calves showed no serologic response to BRSV. Co-infection between BCoV and other agents is likewise possible in FG and EG, as is the case under field conditions.

Unlike most enteric viruses, BCoV is enveloped and therefore susceptible to environmental inactivation [1]. One might expect that the conditions in the forestomaches and abomasum would inactivate BCoV and one possibility is that BCoV is transported from the oronasal cavity to the small intestines through the bloodstream. However, viremia was not detected in the present study, and transport of the virus to the intestines appears to have been through the digestive tract. Park and colleagues [21] detected BCoV RNA in serum samples from calves infected with a winter dysentery strain between day three and eight post inoculation. They used nested PCR for detection, which is generally a more sensitive method than RT-qPCR, but also more vulnerable for contamination [34]. Short viremic period or intake of a lower virus dose in naturally infected calves could also explain the negative results in the present study. Inhibition of the RT-qPCR by plasma components was tested and ruled out. Despite the absence of detectable viremia in the present study, BCoV RNA was found in mesenteric lymph nodes at late stages of the infection. Viral RNA must have been transferred in low concentrations in blood or lymph to the draining lymph node, by antigen presenting cells or as free virus particles.

The finding of BCoV RNA in lymph nodes, ileum and colon six weeks after infection indicates coronavirus persistence in calves, however, the importance of this persistence for virus transmission is uncertain. Other coronaviruses are known to create persistent or chronic infections in mice and cats [35, 36]. MERS-CoV is shown to be excreted for more than a month in humans [37] and human coronavirus 229E creates persistent infections in vitro [38]. Although fecal shedding of BCoV RNA was detected five weeks post infection in the present study, the transmission potential at this stage is most likely negligible, as at three weeks post infection.

BCoV VRC were quantified by RT-qPCR, which does not give information on the number of infective particles. The ratio of total to infective particles (T/I) is challenging to establish for BCoV due to difficulties in cultivating virus from clinical samples. In the present study, virus titration showed a T/I ratio of approximately 5 log_10_. With this high T/I ratio it is not surprising that virus isolation was unsuccessful after D13, when the VRC numbers are decreasing. It also agrees with the sentinel calves not getting infected D21. In contrast, roughly 8.8 log_10_ VRC were detected per nasal swab and gram feces from the seronegative FG calves that infected the EG calves. With a T/I ratio of 5 log_10_, each nasal swab and gram of feces contained more than 3.8 log_10_ infective virus particles.

The high T/I ratio and the failure of virus isolation after D13 could be due to either few infective particles or low sensitivity of the isolation method. Low levels of infective particles could be caused either by high production of defective particles or by neutralizing effect of antibodies. Low sensitivity could be caused by suboptimal conditions in cell culture compared to in vivo (particularly for virus from clinical samples not adapted to cell culture growth), dilution of viral content in the swab, and freezing and thawing of the material. For feline enteric coronavirus, the T/I increased from 3–4 log_10_ during the first week after infection, to up to 8 log_10_ 28 days post infection [39], the increase possibly caused by the antibody response.

Few methods are available for studying transmission potential apart from live animal experiments, although ethically challenging and resource demanding. Existing literature is based on experimental studies examining BCoV shedding for 14 [20, 22, 40] to 21 [19, 41] days. To the authors’ knowledge, the present study is the first to study the shedding for as long as six weeks under experimental conditions. In addition, it is also the first to study the impact of this shedding using sentinel calves. Although a low number of calves were used, the results indicate that calves are not infectious three weeks after exposure to BCoV. This information is important and relevant in order to produce scientific based advices on how to avoid introduction of BCoV into herds. Further investigation of calves at different stages of disease is recommended to verify and corroborate these findings. The effect of stress related to transport on viral shedding and infectivity should also be considered.

In the present study, the virus that caused winter dysentery in adult cattle primarily gave respiratory disease in calves. Niskanen et al. also found that BCoV derived from an outbreak of winter dysentery caused mainly respiratory disease in weaned calves [29], supporting that BCoV is an important cause of respiratory disease in calves [42, 43] and winter dysentery in adults [17]. The economic and welfare consequences of BCoV therefore include the combined effects of neonatal enteritis, respiratory disease in young cattle and winter dysentery in adults. Also considering the high prevalence worldwide, BCoV is an important loss-inflicting factor in the cattle industry.

## Conclusions

The current study shows that calves infected with BCoV are RT-qPCR positive in nasal and fecal specimens for a longer period than earlier recognized. However, contact with naïve calves three weeks after exposure did not lead to infection. A low level of infective particles could be due to either production of a high level of defective particles and/or production of neutralizing antibodies. The study provides highly relevant information when designing biosecurity advice regarding animal trade and coronaviral disease in cattle.

## Additional file

Additional file 1: Table S1. Antibodies to BCoV. (DOCX 14 kb)
