# Supplementary material for: Bovine coronavirus in naturally and experimentally exposed calves; viral shedding and the potential for transmission
Source: Virol J. 2016 Jun 13;13:100. doi: 10.1186/s12985-016-0555-x (PMC4906604; doi:10.1186/s12985-016-0555-x)
Supplement: Additional file 1: Table S1. — Antibodies to BCoV. (DOCX 14 kb) [file 12985_2016_555_MOESM1_ESM.docx]

**Supplementary table S1 – Antibodies to BCoV.**

|  | Day -7 | Day 0 | Day 7 | Day 9 | Day 11 | Day 14 | Day 21 | Day 35 | Day 42 |
| --- | --- | --- | --- | --- | --- | --- | --- | --- | --- |
| F1 | 4 |  |  |  |  | 113 |  |  |  |
| F2 | 1 |  |  |  |  | 93 |  |  |  |
| F3 | 0 |  |  |  |  | 119 |  |  |  |
| F4 | 1 |  |  |  |  | 103 |  |  |  |
| F5 | 1 |  |  |  |  | 93 |  |  |  |
| F6 | 0 |  |  |  |  | 56 |  |  |  |
| E1 |  | -1 | 0 | 3 | 13 | 21 | 60 |  |  |
| E2 |  | 4 | 2 | 7 | 27 | 53 | 80 |  |  |
| E3 |  | 0 | 0 | 1 | 8 | 16 | 70 |  |  |
| E4 |  | 3 | 0 | 3 | 14 | 18 | 90 |  |  |
| S1 |  |  |  |  |  |  | 0/0 | 0 | 2 |
| S2 |  |  |  |  |  |  | 3/0 | 0 | 1 |

Percent positivity (PP) in serum samples analyzed for anti BCoV IgG by ELISA. A PP-value of <10 is regarded negative. The calves were exposed to BCoV in the field (F1-6), were exposed to F-animals day 0 (D0) (E1-4) or exposed to E-animals (S1-2) D21.
